# *Chlamydia pneumoniae* infection of monocytes *in vitro* stimulates innate and adaptive immune responses relevant to those in Alzheimer’s disease

**DOI:** 10.1186/s12974-014-0217-0

**Published:** 2014-12-24

**Authors:** Charles Lim, Christine J Hammond, Susan T Hingley, Brian J Balin

**Affiliations:** Department of Bio-Medical Sciences, Center for Chronic Disorders of Aging, Philadelphia College of Osteopathic Medicine, 4170 City Avenue, Philadelphia, PA 19131 USA

**Keywords:** *Chlamydia pneumoniae*, Inflammation, Innate immunity, Alzheimer’s disease, Gene expression

## Abstract

**Background:**

Alzheimer’s disease (AD) is a progressive neurodegenerative disorder in which infection with *Chlamydia pneumoniae* (Cpn) has been associated. Cpn is an obligate intracellular respiratory pathogen that may enter the central nervous system (CNS) following infection and trafficking of monocytes through the blood-brain barrier. Following this entry, these cells may secrete pro-inflammatory cytokines and chemokines that have been identified in the AD brain, which have been thought to contribute to AD neurodegeneration. The objectives of this work were: (i) to determine if Cpn infection influences monocyte gene transcript expression at 48 hours post-infection and (ii) to analyze whether pro-inflammatory cytokines are produced and secreted from these cells over 24 to 120 hours post-infection.

**Methods:**

Gene transcription was analyzed by RT-PCR using an innate and adaptive immunity microarray with 84 genes organized into 5 functional categories: inflammatory response, host defense against bacteria, antibacterial humoral response, septic shock, and cytokines, chemokines and their receptors. Statistical analysis of the results was performed using the Student's *t*-test. *P*-values ≤ 0.05 were considered to be significant. ELISA was performed on supernatants from uninfected and Cpn-infected THP1 monocytes followed by statistical analysis with ANOVA.

**Results:**

When Cpn-infected THP1 human monocytes were compared to control uninfected monocytes at 48 hours post-infection, 17 genes were found to have a significant 4-fold or greater expression, and no gene expression was found to be down-regulated. Furthermore, cytokine secretion (IL-1β, IL-6, IL-8) appears to be maintained for an extended period of infection.

**Conclusions:**

Utilizing RT-PCR and ELISA techniques, our data demonstrate that Cpn infection of THP1 human monocytes promotes an innate immune response and suggests a potential role in the initiation of inflammation in sporadic/late-onset Alzheimer’s disease.

## Background

Studies from our laboratory have implicated infection with *Chlamydia pneumoniae* (Cpn) in the pathogenesis of sporadic late-onset Alzheimer’s disease (LOAD) [1-3]. We have previously identified infection within monocytes, macrophages, microglia, astroglia, and neurons in the Alzheimer's disease (AD) brain. One mechanism by which the organism may gain access into the brain is following peripheral infection of monocytes [4,5]. We are investigating Cpn infection of monocytes *in vitro* to determine how infection may promote changes in inflammatory gene and protein expression because monocytes have been demonstrated to be altered with regards to expression of cytokines, Aβ amyloid clearance, and apoptosis in Alzheimer's disease patients [6-9].

Although the specific etiology of LOAD has remained elusive, what has emerged is strong evidence that inflammation is a focal point in the neuropathogenesis process [10-13]. Associations between AD and many inflammatory biomarkers, including IL-1β, IL-2, IL-4, IL-6, IL-8, IL-10, IL-12, IL-18, IFN-γ, TNF-α, tumor growth factor beta (TGF-β), and C-reactive protein (CRP) have all been well documented [14] (for reviews see [12,14]). Activated microglia and astroglia have been identified near and around neuritic senile plaques in the AD brain along with pro-inflammatory cytokines IL-1β, IL-6, and TNF-α [11,13,15,16], suggesting that a pro-inflammatory state could be responsible for neurotoxicity associated with AD [17].

As the primary immune cells within the human brain, microglia act as the functional equivalent of macrophages inside the central nervous system (CNS) [18]. Microglia are activated in response to an infection or injury within the brain and are speculated to be responsible for neurotoxicity in diseases such as multiple sclerosis and Parkinson’s disease [18,19]. Experiments in rats have shown that microglia phagocytize and internalize amyloid, apparently in an effort to clear amyloid from the CNS [20]. Related data also indicate that following phagocytosis, Aβ amyloid may remain stored and un-degraded within the activated microglia [21].

Microglial activation as a consequence of internalization of Aβ may activate neighboring microglia and astrocytes through a 'bystander effect'. These activated glia, in turn, may promote further Aβ 1-40 and 1-42 production [22]. This cycle appears to perpetuate inflammation, as well as Aβ production and deposition [10,23]. Activated microglia release a pool of pro-inflammatory factors including IL-1β, IL-6, TNF-α, nitric oxide, IL-8, and macrophage inflammatory protein-1 [24,25]. As IL-1β and IL-6 are major pro-inflammatory cytokines involved with neuronal dysfunction, glia producing these cytokines may start a self-activating cascade referred to as a lethal 'cytokine cycle' [10,11,25]. According to the cytokine cycle, IL-1 is released from activated glia across AD brain regions independent of the initial stimulus. This primary event drives the cascade of cytokine release which promotes the neuropathological changes associated with AD.

A correlation between activated astroglia and amyloid plaques appears to exist, as astroglia, which greatly outnumber microglia in the brain, often aggregate at the site of Aβ deposits [26,27]. Substantial evidence indicates that astroglia are directly involved in the degradation and clearance of Aβ [26], and when activated, they also promote inflammation by secreting IL-1β and IL-6 [28]. These pro-inflammatory mediators also have been shown to activate astroglia resulting in an increased production of Aβ 1-40/1-42 peptides, thus demonstrating a positive feedback loop for the generation of amyloid [23,29]. Chronic glial activation may perpetuate a chain reaction where successive axonal target regions fall victim to additional neuronal damage, thereby continuing the cycle [11].

Furthermore, astroglia have been shown to generate other pro-inflammatory mediators such as monocyte chemoattractant peptide-1 (MCP1/CCL2), RANTES, and TNF-α in reaction to the presence of Aβ [30]. CCL2 is a potent chemoattractant molecule for monocytes, and thus, monocytes may be both initiators and perpetuators of the cycle, suggesting that monocytes could be significant contributors to the pro-inflammatory cascade.

The current study addresses infection of human THP1 monocytes *in vitro* with the laboratory strain of Cpn, AR39, to determine how infection can affect the regulation of gene transcripts for innate and adaptive immunity. In this regard, infection may be a stimulus for pro-inflammatory cytokine expression which may start the cascade that results in initial amyloid processing and deposition observed in Alzheimer's disease [1,31,32]. Analysis of this cytokine expression will demonstrate how infection of monocytes could set the stage for initiating inflammation in this disease.

## Materials and methods

### THP1 human monocytes

THP1 human monocytes, obtained from the American Type Culture Collection (ATCC, Manassas, VA, USA), were propagated at a concentration of 1 × 10^6^ cells/ml RPMI 1640 (ATCC) + 10% FBS (Cellgro Thermo Fisher Scientific, Pittsburgh, PA, USA) growth media (GM) in a 37°C incubator with 5% CO_2_.

### Infection with *Chlamydia pneumoniae* (Cpn)

A human respiratory isolate of Cpn, laboratory strain AR39 obtained from the ATCC, was used to infect THP1 monocytes *in vitro*. Prior to inoculation of monocytes, each vial of Cpn was thawed and sonicated for 2 minutes. THP1 cells were spun down, washed with Hank’s Balanced Salt Solution (HBSS), and resuspended in 1 ml of GM at a concentration of 1 × 10^6^ cells/ml. To this volume, 400 μl of Cpn at a multiplicity of infection (MOI) of 1 (1 × 10^6^ infectious units) were added. The cells were allowed to incubate for 1 hour at 37°C with 5% CO_2_ in a T-25 flask, after which 3.6 ml of GM was added for a final volume of 5 ml. Cells were then incubated for the allotted times (24 to 120 hours). For 96- and 120-hour infections, another 5 ml of GM was added at the 48-hour time point.

To validate and determine percent of infection of our monocytes at each time point, 2 × 10^5^ cells at 1 × 10^5^ cells per slide were cytospun and analyzed by immunocytochemistry. In brief, cytospun cells were fixed using Cytofix/Cytoperm (BD Cytofix/Cytoperm 554722; BD Biosciences, San Jose, CA, USA) for 30 minutes, washed 2 × 5 minutes with HBSS, labeled with a fluorescein isothiocyanate (FITC)-directly conjugated anti-chlamydial antibody, Fitzgerald 61C75 (Fitzgerald Industries International, Acton, MA, USA) for 1 hour at 37°C, and counterstained using bisbenzimide (Sigma bisbenzimide B2883, Sigma-Aldrich, St Louis, MO, USA) and coverslipped. The slides were imaged on a Nikon E80i microscope using the NIS-Elements AR 3.0 software (Nikon Inc., Melville, NY, USA). Five random fields per slide per time point for each experiment at 40X magnification were analyzed and counted (total infected cells/total cells × 100 = percent infected). Average percent infection for 24 to 120 hours was 81, 85, 92, 92, and 90, respectively.

Infection time for the real time-polymerase chain reaction (RT-PCR) experiments was 48 hours and the infection times for the ELISA cytokine analyses were 24 to 120 hours. Although gene changes were analyzed only at 48 hours post-infection, the time at which an acute infection is established, we wanted to obtain specific data for cytokine changes over longer time points as the inflammation in AD appears to become more chronic in nature following its initiation. In a comparable manner, parallel control uninfected flasks at the same cellular concentration received only media for the specified incubation times.

At the specified times for analysis, both uninfected and infected cells were removed from the incubator and transferred to individual 15-ml tubes. To ensure complete cell recovery, another 5 ml of fresh GM was added to the 24-, 48- and 72-hour flasks, followed by cell scraping to release any remaining cells prior to transfer to the 15-ml tubes. The final volume for all time points was 10 ml. The cells were pelleted at 1,000 x g for 5 minutes. The supernatants were collected for ELISA analysis into new 15-ml tubes and frozen at −80°C. The pellet was resuspended in 5 ml of HBSS and counted. Cells were again pelleted and either kept on ice or snap frozen in liquid nitrogen prior to preparation for RT-PCR.

### RNA isolation

Using the RNeasy Mini kit (Qiagen Inc., Valencia, CA, USA), RNA was extracted from cell pellets according to manufacturer’s directions. In brief, cells were mixed with RLT buffer, pipetted onto a QIAshredder™ spin column and centrifuged for 2 minutes at 15,000 × g to lyse and homogenize the cells. Ethanol was mixed with this sample, transferred to an RNeasy spin column, centrifuged for 15 seconds at 8,000 × g and the flow-through discarded. RW1 Buffer (700 μl) was added, centrifuged for 15 seconds at 8,000 × g and flow-through discarded. RPE Buffer (500 μl) was added to the RNeasy spin column, centrifuged for 15 seconds at 8,000 × g, flow-through discarded, another 500 μl of RPE Buffer was added and centrifuged for 2 minutes at 8,000 × g. Finally, the RNA was eluted from the spin column in RNase-free water (30 μl).

### First strand synthesis

Using the RT^2^ First Strand Kit (SABiosciences, Qiagen, Valencia, CA, USA), cDNA was produced from 1 μg of RNA following the First Strand Kit protocol according to manufacturer’s directions. To eliminate any contaminating genomic DNA carryover, a Genomic DNA Elimination step preceded the reverse transcription reaction. Following reverse transcription, 91 μl of RNase, DNase-free water was added to each 20 μl of cDNA. This cDNA was either used immediately for RT-PCR or frozen at −20°C until use.

### Real time-polymerase chain reaction (RT-PCR)

RT-PCR was used to determine the level of gene transcription using the Human Innate and Adaptive Immune Responses RT^2^ Profiler™ PCR Array (PAHS-052) from SABiosciences (Qiagen, Valencia, CA, USA). The cDNA samples were spun briefly to remove any particulate material. The master mix cocktail was prepared according to the specifications for the 96-well plate.

Twenty-five microliters of the experimental cocktail was added to each well of the 96-well RT-PCR array plate. The plate was run on the Applied Biosciences 7000 Sequence Detection System (Life Technologies Corporation, Carlsbad, CA, USA) utilizing ABI Prism 7000 SDS Software and then analyzed using software on the SABiosciences website [33]. The data are normalized to the control housekeeping genes *RPL13A*, *GAPDH* and *ACTB*. The website calculates fold change (fold difference) by the 2^-ΔΔ^CT method with the control being the uninfected THP1 cells. Statistical analysis of the results was done using Student’s *t*-test. *P*-values below 0.05 were considered to be significant. RT-PCR experiments were performed using cDNA from 4 separate experiments comparing 48 hour Cpn-infected THP1 monocytes to parallel uninfected monocytes grown for 48 hours.

### Multi-analyte ELISA array

Using the Multi-Analyte ELISArray Kit protocol version 1.4 from SABiosciences (Qiagen, Valencia, CA, USA), a multi-array ELISA was performed using the supernatants collected during the harvest of uninfected and Cpn-infected THP1 monocytes according to manufacturer’s directions. In brief, the supernatants were thawed and centrifuged for 10 minutes at 1,000 × g to remove any particulate material. Fifty microliters of each experimental sample, in triplicate, was added to the array that included specific cytokine capture antibodies: IL-1α IL-1β, IL-2, IL-4, IL-6, IL-8, IL-12, IL-17α, IFN-γ, TNF-α and GM CSF (colony stimulating factor) and allowed to incubate at room temperature (RT) for 2 hours.

After numerous buffer washes, 100 μl of the diluted biotinylated detection antibodies were added to the appropriate wells of the ELISA plate and incubated in the dark for 1 hour at room temperature (RT). Following this incubation, the plate was washed and 100 μl of dilute Avidin-horseradish peroxidase (HRP) were added into all wells and incubated in the dark for 30 minutes at RT. After this incubation, development and stop solutions were added followed by detection of absorbance changes at 450 and 570 nm on a UV/VIS spectrophotometer (Nicolet Evolution 100, Thermo Fisher Scientific, Pittsburgh, PA, USA).

The raw data obtained from the absorbance readings were normalized to the cell counts of each sample. Averages of all triplicates were determined along with standard deviations and an analysis of variance (ANOVA) statistical test was performed. Time points of 24, 48 and 72 hours were tested in triplicate in 5 separate experiments, while the 96- and 120-hour time points were tested in triplicate in 4 separate experiments.

### Single-analyte ELISA array

Using the Single-Analyte ELISArray Kit protocol version 1.4 from SABiosciences (Qiagen, Valencia, CA, USA), a single-analyte ELISA was performed for IL-1β, IL-6, and IL-8 according to manufacturer’s directions using the supernatants collected during the harvest. In brief, the supernatants were thawed and centrifuged for 10 minutes at 1,000 × g to remove any particulate material. Preparation of the antigen standard dilution was prepared by serial dilution. As with the multi-analyte array, the array was prepared using 50 μl of each experimental sample in triplicate and allowed to incubate at RT for 2 hours, followed by incubation with the detection antibodies. Similar to the previous multi-analyte arrays, Avidin-HRP was used to determine binding of the detection antibodies to the cytokines of interest. After color development as described above, absorbance readings were taken at 450 and 570 nm and the raw data were normalized. Averages of all triplicates were found along with standard deviations and an ANOVA statistical test was performed. As with the multi-analyte array, times of 24, 48 and 72 hours were tested in 5 separate experiments and those of 96 and 120 hours were tested from 4 separate experiments.

## Results

### Real time-polymerase chain reaction

Gene transcription was analyzed by RT-PCR using an innate and adaptive immunity microarray with 84 genes organized into 5 functional categories: inflammatory response, septic shock, cytokines, chemokines and their receptors, host defense against bacteria, and antibacterial humoral response. When Cpn-infected THP1 human monocytes were compared to control uninfected monocytes at 48 hours post-infection, 17 gene transcripts (Table 1) had significant increases (*P*-values ≤ 0.05).

**Table 1:** **Innate and adaptive immunity gene transcripts increased at 48 hours in** ***Chlamydia pneumoniae*** **(Cpn)-infected THP1 cells**

	**Gene symbol**	**Gene name**
Inflammatory response	*IL1F5*	Interleukin 1 family, member 5 (delta)
	*IL1F8*	Interleukin 1 family, member 8 (eta)
	*IL1RN*	Interleukin 1 receptor antagonist
	*IRAK2*	Interleukin 1 receptor associated kinase 2
	*NLRC4*	NLR family, CARD domain containing 4
	*TLR8*	Toll-like receptor 8
	*TNF*	Tumor necrosis factor (TNF superfamily, member 2)
Host defense against bacteria	*DEFB4*	Defensin, beta 4
	*DMBT1*	Deleted in malignant brain tumors 1
	*NFKBIA*	Nuclear factor of kappa light polypeptide gene enhancer in B-cells inhibitor, alpha
	*PTAFR*	Platelet-activating factor receptor
Antibacterial response	*COLEC12*	Collection sub-family member 12
	*CYBB*	Cytochrome B-245, beta polypeptide
Cytokines, chemokines, and their receptors	*CCL2*	Chemokine (C-C motif) ligand 2
	*IFNB1*	Interferon, beta 1, fibroblast
	*IL6*	Interleukin 6 (Interferon, beta 2)
Septic shock	*SERPINA1*	Serpin peptidase inhibitor, clade A (alpha-1 antiproteinase, antitrypsin), member 1

Transcripts analyzed included those associated with the host response to pathogens, including inflammatory response genes, antibacterial humoral response genes, cytokines, chemokines and their receptors, genes involved in bacterial host defense mechanisms and septic shock.

**Figure 1:**
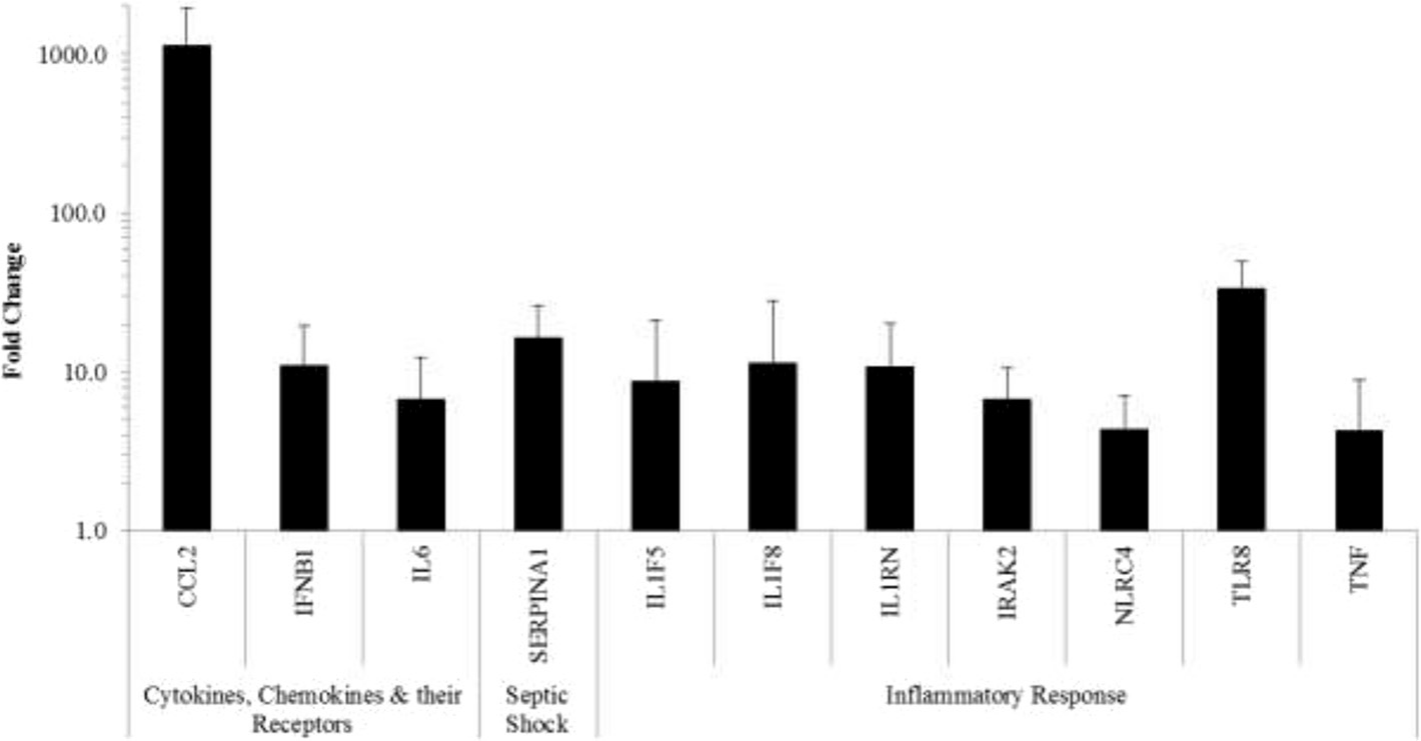
**Inflammatory response, cytokines, chemokines and their receptors, and septic shock gene transcripts.** Transcript increases of THP1 human monocytes at 48 hours post Cpn infection. Only significant gene transcripts with a 4-fold or greater increase compared to uninfected THP1 cells are included; data are plotted on a log scale (*P*-values ≤ 0.05).

Seven gene transcripts from the inflammatory response category (see Table 1 and Figure 1) had at least a four-fold increase in expression. Toll-like receptor (*TLR*)*8* transcripts had the most dramatic significant fold increase of 33.73, whereas lesser increases were observed for *TNF* (4.26), *NLRC4* (4.34), *IL1F5* (8.79), *IL1F8* (11.29), and the IL-1 receptor genes *IL1RN* (10.83) and *IRAK2* (6.66).

Gene transcripts for 3 cytokines, chemokines and their receptors demonstrated significant > 4-fold increases (Figure 1). *CCL2* transcripts had the most dramatic significant fold increase of 1,136.10 within this category. Expression of *IFNB1* and IL-6 both had significant increases of 11.02 and 6.69, respectively. Under septic shock (Figure 1), only 1 gene (*SERPINA1*, 16.50) was found to have a significant increase in expression.

**Figure 2:**
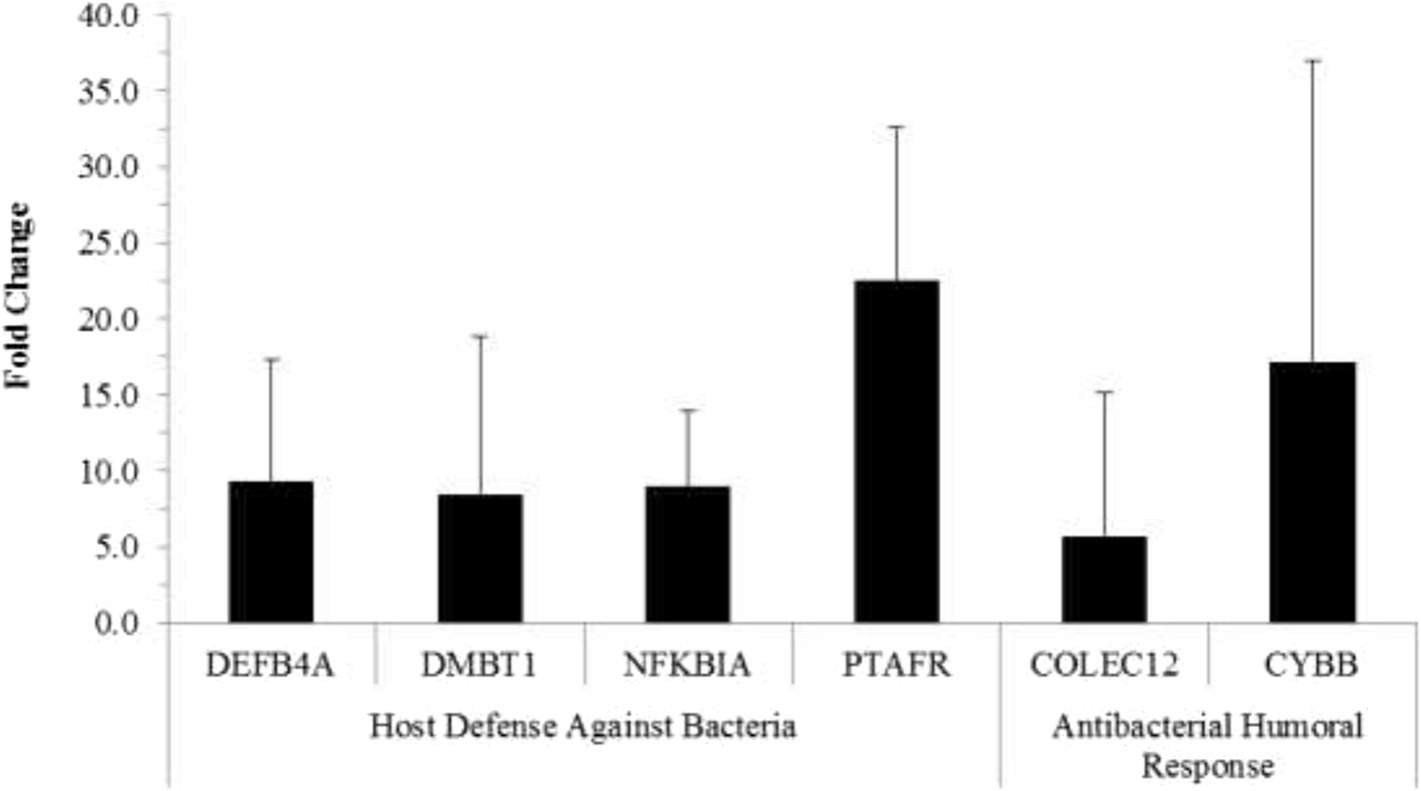
**Host defense against bacteria and antibacterial humoral response gene transcripts.** Gene transcript increases of THP1 human monocytes at 48 hours post Cpn infection. Only significant gene transcripts with a 4-fold or greater increase compared to uninfected THP1 cells are included; (*P*-values ≤ 0.05).

Transcript changes with regard to the host defense against bacteria revealed four gene transcripts to be significantly up-regulated with > four-fold increases (Figure 2). PTAFR had the most dramatic significant increase in expression with 22.47 fold change. Under antibacterial humoral response (Figure 2), *CYBB* gene expression had the most significant increase of 17.15, while *COLEC12* had a significant increase of 5.64.

In summary, of all the 84 genes in the Innate and Adaptive Immunity pathway microarray, expression of *CCL2* showed the most dramatic significant increase. Other cytokine gene transcripts of note such as *IL-6* and *TNF* showed smaller, but significant fold increases in expression.

### ELISA

ELISA techniques were performed using the supernatants of uninfected and Cpn-infected THP1 human monocytes. A multi-analyte ELISArray provided a general overview for secretion per 1 × 10^6^ cells of 12 cytokines. This ELISArray showed that the cytokines IL-1β, IL-6, and IL-8 have increased secretion when the cells are Cpn-infected, thus single-analyte ELISArrays were performed to further evaluate these 3 cytokines.

### IL-1β

**Figure 3:**
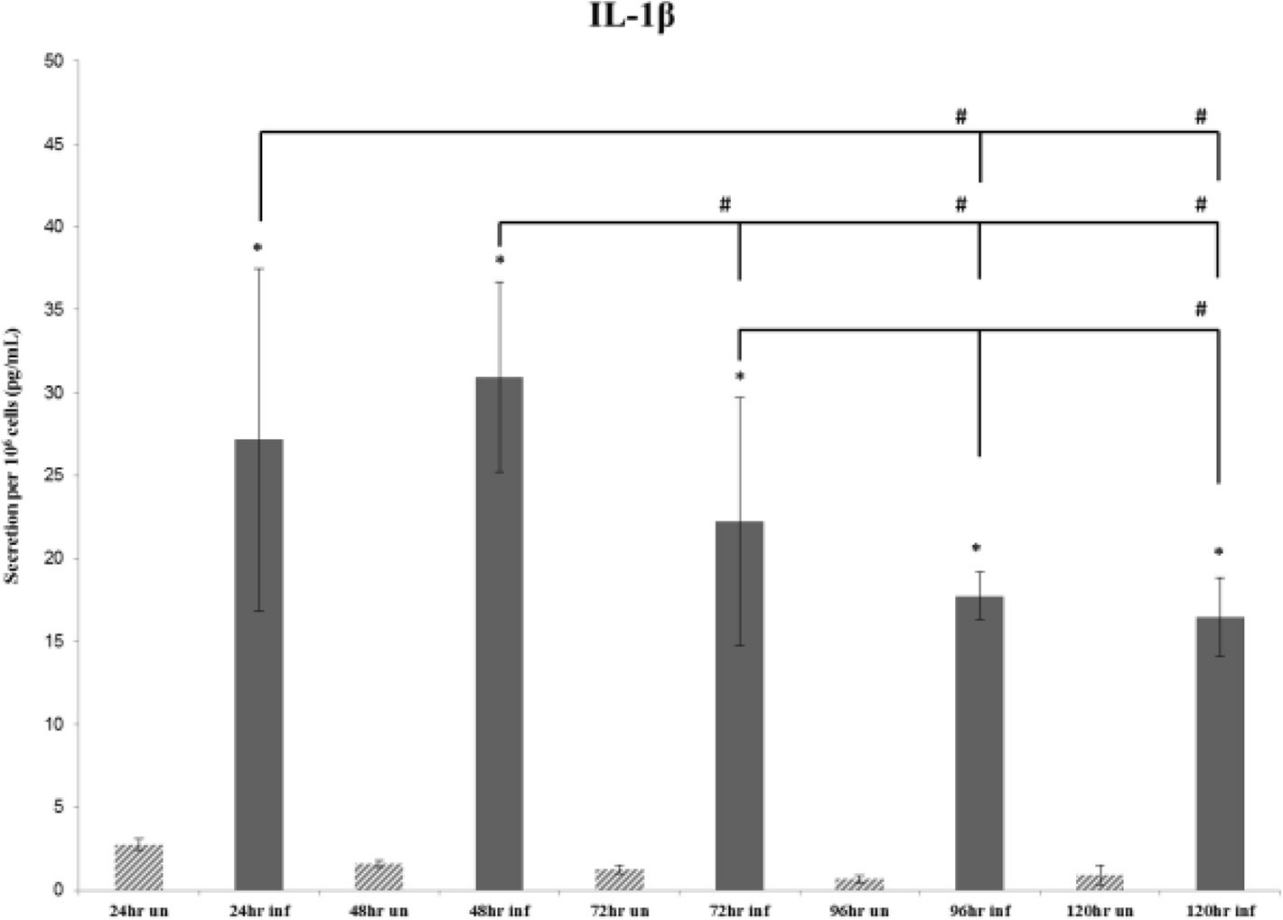
**Average secretion of IL-1β.** Average secretion of IL-1β by 10^6^ cells at the 24-, 48- and 72-hour time points repeated in 5 individual experiments, and the 96 hours and 120 hours repeated in 4 individual experiments. The (*) symbol indicates significance (*P*-value ≤ 0.05) within time points (uninfected to infected), while the (#) symbol indicates significance (*P*-value ≤ 0.05) between time points (infected to infected).

A single-analyte ELISArray for IL-1β (Figure 3) demonstrated a significant increase (*P*-value ≤ 0.05) in cytokine secretion in the Cpn-infected THP1 human monocytes compared to uninfected THP1 cells for all the time points. In Cpn-infected THP-1 cells, a significant decrease in levels of secreted IL-1β at the 96-hour and 120-hour time points relative to the 24-hour time point was observed. Decreased secretion of IL-1β also was significant for the Cpn-infected THP1 cells at the 72-, 96- and 120-hour time points compared to the levels at 48 hours. At 120 hours, there was significantly lower secretion of IL-1β compared to 72 hours.

### IL-6

**Figure 4:**
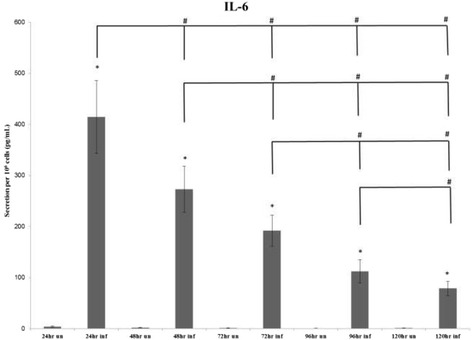
**Average secretion of IL-6.** Average secretion of IL-6 by 10^6^ cells at the 24-, 48- and 72-hour time points repeated in 5 individual experiments and the 96 hours and 120 hours repeated in 4 individual experiments. The (*) symbol indicates significance (*P*-value ≤ 0.05) within time points (uninfected to infected), while the (#) symbol indicates significance (*P*-value ≤ 0.05) between time points (infected to infected).

Analysis of the IL-6 single-analyte ELISArray plate revealed that more IL-6 is secreted from Cpn-infected THP1 cells than uninfected THP1 cells at all times (Figure 4); this difference was significant. Moreover, there was an overall significant decrease in IL-6 secretion from Cpn-infected THP1 cells over the entire time course of infection.

### IL-8

**Figure 5:**
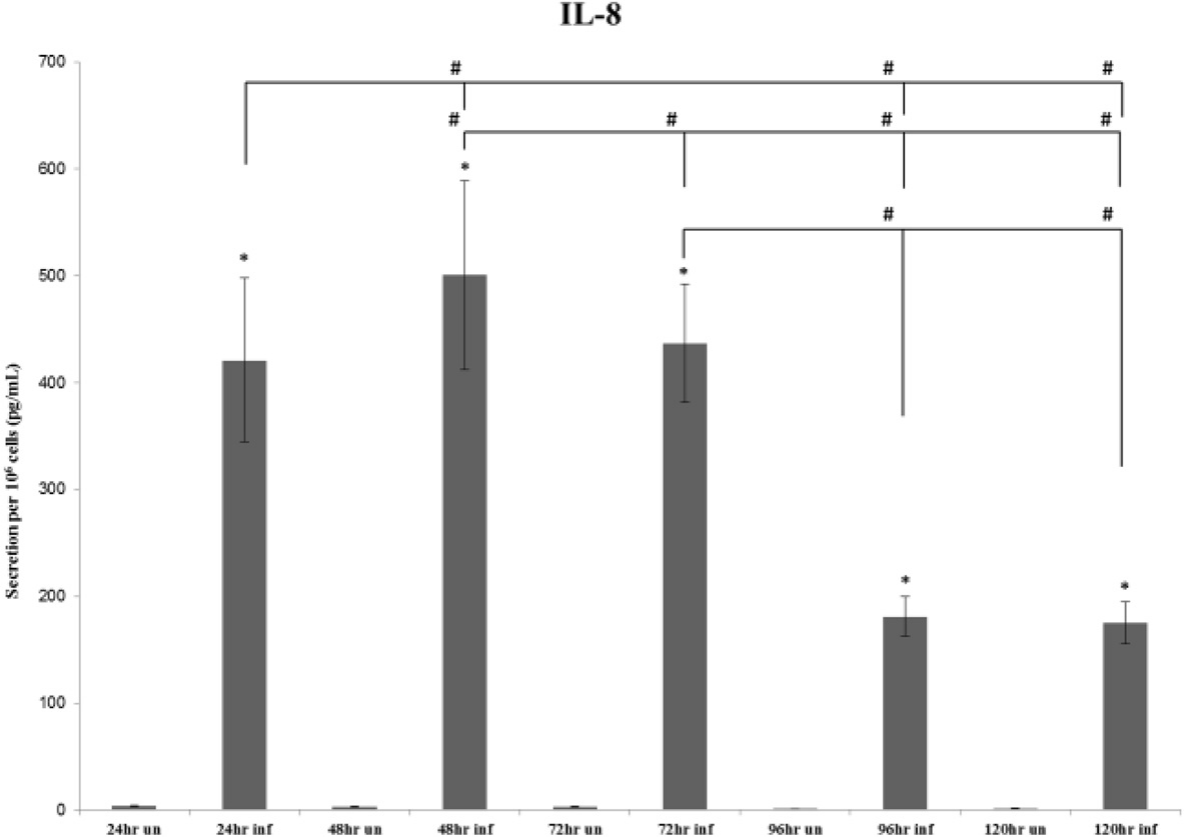
**Average secretion of IL-8.** Average secretion of IL-8 by 10^6^ cells at the 24-, 48- and 72-hour time points repeated in 5 individual experiments and the 96 hours and 120 hours repeated in 4 individual experiments. The (*) symbol indicates significance (*P*-value ≤ 0.05) within time points (uninfected to infected), while the (#) symbol indicates significance (*P*-value ≤ 0.05) between time points (infected to infected).

Results of the IL-8 single-analyte ELISArray plate revealed an initial significant increase (*P*-value ≤ 0.05) in the 24-, 48-, 72-, 96- and 120-hour time points between Cpn-infected cells compared to uninfected THP1 (Figure 5). Similar to what was observed with IL-1β, at 96 and 120 hours, there was a marked decrease in secretion of IL-8 from the Cpn-infected THP1 cells compared to the Cpn-infected cells at earlier time points. Furthermore, the decreases seen at 96 and 120 hours as compared to 24 and 48 hours were significant (*P*-value ≤ 0.05), as was the decrease seen from the 48-hour time to the 72-hour time point, and the decrease seen from 72 hours to the 96- and 120-hour time points.

## Discussion

Chlamydial species have been associated with a spectrum of diseases of clinical significance shown to be a result of immunopathogenesis. Although the immune response is central for disease manifestations, there is still much to learn about the mechanisms that initiate and sustain the inflammatory response to Cpn infection [34,35]. Secretion of pro-inflammatory cytokines occurs when host cells, including monocytes and microglia, are infected with Cpn [35,36]. The current study evaluates changes in THP1 monocyte gene expression at 48 hours post-infection (acute) and in specific cytokine production/secretion over a 5-day (chronic) course of infection. Our data indicate that infection of monocytes with Cpn results in significant changes in host gene transcription and secretion of pro-inflammatory cytokines. Furthermore, cytokine secretion (IL-1β, IL-6, IL-8) appears to be maintained for an extended period of infection, suggesting that a more chronic or persistent infection can maintain the pro-inflammatory condition. Work by others has suggested that similar cytokines were secreted after Cpn infection of THP1 monocytes, although shorter periods of infection with a higher multiplicity of infection (ie, MOI = 5) were evaluated [37]. Thus, our data suggest that Cpn-infected monocytes at a modest level of infection (ie, MOI = 1) can promote an acute and sustained pro-inflammatory state.

Previous work has shown that Cpn can infect multiple cell types including endothelial cells, epithelial cells, smooth muscle cells, and peripheral blood mononuclear cells [1,38,39]. Cpn-infected macrophages have been found in sites such as the peritoneum and spleen following respiratory inoculation of New Zealand white rabbits [40]. These findings suggest that the migratory capabilities of peripheral blood monocytes may help to facilitate systemic infection [41]. Moreover, infection of human monocytes with Cpn appears to enhance their migration across an *in vitro* model of the blood-brain barrier (BBB) comprised of a human brain microvascular endothelial cell layer [4]. Previous studies have shown that infection in the lung is the main stimulus for monocytic and lymphocytic infiltration leading to potential dissemination of infection into the circulation [40,42]. Blood vessels and monocytes in AD brain tissue have been shown to be infected with Cpn, thus we postulate that Cpn entering the circulation upon infection of monocytes could result in access to the CNS through the BBB [4,5,43]. With regards to LOAD, Cpn has been studied as a possible etiologic agent [1,2] and supporting evidence for Cpn in the etiopathogenesis of AD was provided by a study by Little *et al*. 2004 which revealed amyloid deposits resembling plaques found in AD brains in the brains of non-transgenic BALB/c mice following intranasal infection with Cpn [42].

As discussed previously, numerous cytokines have been shown to be involved in neuroinflammation in AD, and previous work by others has implicated high levels of IL-1 directly in neuronal degeneration [44,45]. IL-1β is a member of the interleukin 1 cytokine family and is produced by endothelial cells, some epithelial cells, and also by activated macrophages as a pro-protein, which is proteolytically processed to its active form by IL-1β convertase. This cytokine is an important mediator of the inflammatory response, and is involved in a variety of cellular activities, including cell proliferation, differentiation, and apoptosis [46]. Also, IL-1β activates nitric oxide synthase (NOS), which is implicated in neuronal damage, ultimately leading to cell death in hippocampal neurons [47]. Furthermore, IL-1β has been shown to play a part in the promotion of neuronal synthesis of β amyloid precursor protein (APP) and may enhance the transition from diffuse to fibrillar neuritic plaques [48]. Our data suggest that infection may play an early role in the etiology of AD, even before amyloid is produced. This contrasts with others who have focused previously on amyloid as being the principal stimulus for the activation of this cytokine [28].

The key debate, therefore, lies not in what this cytokine can activate and/or damage in the brain, but rather what initiates its production. Levels of IL-1β have been shown to be higher in the cerebrospinal fluid (CSF) of AD patients than in patients with vascular dementia or control patients [49,50]. Our data show that IL-1β is significantly up-regulated at all times through 120 hours post-infection of monocytes and thus is consistent with the cytokine profile in AD, suggesting that infection can be an initiating stimulus for this cytokine.

Evidence suggests that IL-1β and IL-6 up-regulate the cdk5/p35 complex [51], a protein kinase involved in *tau* hyperphosphorylation, a major pathological process observed in AD. IL-6 also may be involved in neuronal degradation. IL-6 is an inflammatory cytokine that is secreted by leukocytes and other various cell types during infection [52]. As a pro-inflammatory cytokine, IL-6 is secreted by T-cells, endothelial cells, and macrophages, which stimulate an immune response to tissue damage leading to inflammation in the surrounding area. IL-6 is an important mediator of fever and of the acute phase response. IL-6 can be secreted by macrophages in response to specific microbial molecules, referred to as pathogen associated molecular patterns (PAMPs), and can induce intracellular signaling cascades that can further give rise to inflammatory cytokine production [53]. Infection with Cpn, as we have determined in this study, influences significant secretion of this cytokine from infected monocytes.

Not surprisingly, in our study, several gene transcripts were found to have significant fold increases in the group consisting of the host defense against bacteria. These included: *DEFB4*, *DMBT1*, *PTAFR*, and *NFKBIA. DEFB4* is the transcript that codes for a defensin protein with antimicrobial activity that has been shown to act as a cytokine linking innate and adaptive immune responses [54]. Along these lines, Aβ amyloid 1-42 has been demonstrated to have antibacterial properties and has been speculated to act as an anionic defensin [55]. However, whether Aβ 1-42 production and processing in LOAD results, in part, as an antibacterial response is still debatable. Therefore, defensin transcript increases are intriguing and warrant further study.

Up-regulation of *DMBT1* takes place in response to activation of the intracellular pattern recognition molecule NOD2 and consecutive NFkB-activation; this hinders bacterial invasion and lipopolysaccharide (LPS)-induced TLR4 activation [56]. *PTAFR* is the gene for the platelet activating factor receptor, which plays a role in the inflammatory response [57]. *NFKBIA* could lead to inhibition of the NFκB protein complex by trapping this transcription factor in the cytoplasm, effectively leading to its inactivation. However, as NFκB activation is upstream of gene transcription for IL-1β mentioned above, its complete inactivation by *NFKBIA* up-regulation appears unlikely in our infected cells.

Another interesting entity found to be activated in infected cells is the inflammasome complex. This is a cytoplasmic multi-protein complex that activates caspase-1, leading to the processing and secretion of pro-inflammatory cytokines. These complexes are associated with TLRs to mediate the response to extracellular and intracellular pathogens [58]. *NLRC4* (*NLR family*, *CARD domain containing 4*) is involved in the regulation of caspase-1, which upon activation results in processing and secretion of IL-1β. In this study, the *NLRC4* gene transcript showed significant fold increases. Previously, others have shown that *NLRC4* is required for the activation of caspase-1 and IL-1β secretion in response to bacterial flagellin [59]. Interestingly, inflammasome activation also may arise as an outcome of amyloid deposition and aggregation [60]. By orchestrating the activation of precursors of pro-inflammatory caspases, which in turn activate IL-1β, IL-18 and IL-33 secretion, the inflammasome promotes a potent inflammatory response that influences the release of toxins from glial and endothelial cells [61]. Although the NLRC4 inflammasome has been shown to be activated by flagellated Gram-negative bacteria, recent studies have shown evidence for a flagellin-independent pathway that activates the NLRC4 inflammasome after infection with certain aflagellated bacteria [62]. As Cpn is an intracellular bacterium and aflagellated, our data would support that of a flagellin-independent pathway. Another important response to infection is one in which acute phase reactants are produced and secreted into blood. Of these, serpins are included and constitute the largest superfamily of protease inhibitors in humans [63,64]. In the microarray used in this study, SerpinA1 (Serpin peptidase inhibitor clade A member 1) transcript was shown to increase significantly following infection. This serpin is also known as α_1_-antitrypsin (AAT), a highly effective inhibitor of neutrophil elastase and one that plays an important role in coagulation, inflammation, and turnover of extracellular matrix. SerpinA1 also inhibits the activity of plasmin, thrombin, trypsin, chymotrypsin, and plasminogen activator.

*MCP1*/*CCL2* gene transcript in the family of cytokines and chemokines was significantly increased following Cpn infection. This transcript was the most dramatically altered following infection and has been recognized as a very important contributor to the inflammatory response observed in AD [65]. The MCP1/CCL2 protein has been shown to be increased in both CSF and plasma from individuals with mild cognitive impairment (MCI) and AD [66]. While the exact effects of MCP1/CCL2 in the MCI/AD brain are not fully understood, some experiments have shown that CCL2 may alter the properties of the BBB to allow for increased monocyte migration into brain tissues [67]. Furthermore, this chemokine may affect the production and clearance of Aβ amyloid from the brain [68]. Hence, although we did not measure the protein directly, the dramatic increase in this transcript would suggest that infection induction leading to such a large change is worthy of further evaluation.

## Conclusion

Utilizing RT-PCR and ELISA techniques, our data demonstrate that Cpn infection of THP1 human monocytes promotes an innate immune response, as pro-inflammatory gene transcripts and proteins showed significant fold increases. Furthermore, since research suggests that a chronic inflammatory state is present within the AD brain [10] and prior evidence has shown monocytes infected with Cpn in AD brains [1,3], we suggest that the pro- and chronic inflammatory states involved in AD pathogenesis may arise in part by Cpn infection of monocytes. These data are consistent with that of previous work suggesting that amyloid could be both a response to and an initiator of inflammation in the AD brain [22]. In effect, infection in the AD brain could initiate the inflammatory cascade that results in CNS damage reflected by amyloid production/processing and deposition. Inflammation generated by amyloid accumulation would then 'secondarily' result in extended damage via an inflammatory response generating ever increasing AD neuropathology. Further research to define the early events occurring in AD pathogenesis may help to clarify how infection with Cpn, and possibly other pathogens, may be an initiator of inflammation in sporadic LOAD.

## Abbreviations

AAT: α1-antitrypsin
Aβ: β-amyloid
ABI: Applied BIosystems
AD: Alzheimer’s disease
ANOVA: analysis of variance
APP: amyloid precursor protein
ATCC: American type culture collection
BBB: blood-brain barrier
cDNA: complementary deoxyribonucleic acid
Cpn: *Chlamydia pneumoniae*
CNS: central nervous system
CRP: C-reactive protein
CSF: cerebrospinal fluid
CT: cycle threshold
ELISA: enzyme linked immunosorbent assay
FBS: fetal bovine serum
FITC: fluorescein isothiocyanate
GM: growth media
HBSS: Hanks balanced salt solution
HRP: horseradish peroxidase
ie: that is
IFN: interferon
IL: interleukin
LOAD: late-onset Alzheimer’s disease
LPS: lipopolysaccharide
MCI: mild cognitive impairment
MCP1: monocyte chemoattractant peptide-1
MOI: multiplicity of infection
NFκB: nuclear factor kappa B
NLRC4: Nod –like receptor family CARD domain-containing protein 4
NOD2: Nucleotide-binding oligomerization domain-containing protein 2
NOS: nitric oxide synthase
PAMPS: pathogen associated molecular patterns
Rantes: regulated on activation normal T cell expressed and secreted
RNA: ribonucleic acid
RPMI: Roswell Park Memorial Institute
RT: room temperature
RT-PCR: real time – polymerase chain reaction
TGF-β: tumor growth factor beta
TLR4: toll-like receptor 4
TNF-α: tumor necrosis factor alpha
UV/VIS: ultraviolet/visible

## References

[1] Balin BJ, Gerard HC, Arking EJ, Appelt DM, Branigan PJ, Abrams JT, Whittum-Hudson JA, Hudson AP (1998). Identification and localization of *Chlamydia pneumoniae* in the Alzheimer’s brain. Med Microbiol Immunol.

[2] Gerard HC, Dreses-Werringloer U, Wildt KS, Deka S, Oszust C, Balin BJ, Frey WH, Bordayo EZ, Whittum-Hudson JA, Hudson AP (2006). *Chlamydophila* (*Chlamydia*) *pneumoniae* in the Alzheimer’s brain. FEMS Immunol Med Microbiol.

[3] Hammond CJ, Hallock LR, Howanski RJ, Appelt DM, Little CS, Balin BJ (2010). Immunohistological detection of *Chlamydia pneumoniae* in the Alzheimer’s disease brain. BMC Neurosci.

[4] MacIntyre A, Abramov R, Hammond CJ, Hudson AP, Arking EJ, Little CS, Appelt DM, Balin BJ (2003). *Chlamydia pneumoniae* infection promotes the transmigration of monocytes through human brain endothelial cells. J Neurosci Res.

[5] MacIntyre A, Hammond CJ, Little CS, Appelt DM, Balin BJ (2002). *Chlamydia pneumoniae* infection alters the junctional complex proteins of human brain microvascular endothelial cells. FEMS Microbiol Lett.

[6] Fiala M, Lin J, Ringman J, Kermani-Arab V, Tsao G, Patel A, Lossinsky AS, Graves MC, Gustavson A, Sayre J, Sofroni E, Suarez T, Chiappelli F, Bernard G (2005). Ineffective phagocytosis of amyloid-beta by macrophages of Alzheimer’s disease patients. J Alzheimers Dis.

[7] Fiala M, Cribbs DH, Rosenthal M, Bernard G (2007). Phagocytosis of amyloid-beta and inflammation: two faces of innate immunity in Alzheimer’s disease. J Alzheimers Dis.

[8] Saresella M, Marventano I, Calabrese E, Piancone F, Rainone V, Gatti A, Alberoni M, Nemni R, Clerici M (2014). A complex proinflammatory role for peripheral monocytes in Alzheimer’s disease. J Alzheimers Dis.

[9] Feng Y, Li L, Sun XH (2011). Monocytes and Alzheimer’s disease. Neurosci Bull.

[10] Ringheim GE, Conant K (2004). Neurodegenerative disease and the neuroimmune axis (Alzheimer’s and Parkinson’s disease, and viral infections). J Neuroimmunol.

[11] Griffin WS, Sheng JG, Royston MC, Gentleman SM, McKenzie JE, Graham DI, Roberts GW, Mrak RE (1998). Glial-neuronal interactions in Alzheimer’s disease: the potential role of a ‘cytokine cycle’ in disease progression. Brain Pathol.

[12] Swardfager W, Lanctot K, Rothenburg L, Wong A, Cappell J, Herrmann N (2010). A meta-analysis of cytokines in Alzheimer’s disease. Biol Psychiatry.

[13] McGeer PL, McGeer EG (2002). Local neuroinflammation and the progression of Alzheimer’s disease. J Neurovirol.

[14] McGeer EG, McGeer PL (2010). Neuroinflammation in Alzheimer’s disease and mild cognitive impairment: a field in its infancy. J Alzheimers Dis.

[15] Akiyama H, Barger S, Barnum S, Bradt B, Bauer J, Cole GM, Cooper NR, Eikelenboom P, Emmerling M, Fiebich BL, Finch CE, Frautschy S, Griffin WS, Hampel H, Hull M, Landreth G, Lue L, Mrak R, Mackenzie IR, McGeer PL, O’Banion MK, Pachter J, Pasinetti G, Plata-Salaman C, Rogers J, Rydel R, Shen Y, Streit W, Strohmeyer R, Tooyoma I, Van Muiswinkel FL, Veerhuis R, Walker D, Webster S, Wegrzyniak B, Wenk G, Wyss-Coray T (2000). Inflammation and Alzheimer’s disease. Neurobiol Aging.

[16] Rogers J, Lue LF (2001). Microglial chemotaxis, activation, and phagocytosis of amyloid beta-peptide as linked phenomena in Alzheimer’s disease. Neurochem Int.

[17] Saez TE, Pehar M, Vargas M, Barbeito L, Maccioni RB (2004). Astrocytic nitric oxide triggers tau hyperphosphorylation in hippocampal neurons. In Vivo.

[18] Lue LF, Walker DG, Rogers J (2001). Modeling microglial activation in Alzheimer’s disease with human postmortem microglial cultures. Neurobiol Aging.

[19] Kreutzberg GW (1996). Microglia: a sensor for pathological events in the CNS. Trends Neurosci.

[20] Frautschy SA, Cole GM, Baird A (1992). Phagocytosis and deposition of vascular beta-amyloid in rat brains injected with Alzheimer beta-amyloid. Am J Pathol.

[21] Frackowiak J, Wisniewski HM, Wegiel J, Merz GS, Iqbal K, Wang KC (1992). Ultrastructure of the microglia that phagocytose amyloid and the microglia that produce beta-amyloid fibrils. Acta Neuropathol.

[22] Bitting L, Naidu A, Cordell B, Murphy GM (1996). Beta-amyloid peptide secretion by a microglial cell line is induced by beta-amyloid-(25–35) and lipopolysaccharide. J Biol Chem.

[23] Blasko I, Stampfer-Kountchev M, Robatscher P, Veerhuis R, Eikelenboom P, Grubeck-Loebenstein B (2004). How chronic inflammation can affect the brain and support the development of Alzheimer’s disease in old age: the role of microglia and astrocytes. Aging Cell.

[24] Lue LF, Rydel R, Brigham EF, Yang LB, Hampel H, Murphy GM, Brachova L, Yan SD, Walker DG, Shen Y, Rogers J (2001). Inflammatory repertoire of Alzheimer’s disease and nondemented elderly microglia *in vitro*. Glia.

[25] Benveniste EN, Nguyen VT, O’Keefe GM (2001). Immunological aspects of microglia: relevance to Alzheimer’s disease. Neurochem Int.

[26] Dickson DW (1997). The pathogenesis of senile plaques. J Neuropathol Exp Neurol.

[27] Savchenko VL, McKanna JA, Nikonenko IR, Skibo GG (2000). Microglia and astrocytes in the adult rat brain: comparative immunocytochemical analysis demonstrates the efficacy of lipocortin 1 immunoreactivity. Neuroscience.

[28] Benzing WC, Wujek JR, Ward EK, Shaffer D, Ashe KH, Younkin SG, Brunden KR (1999). Evidence for glial-mediated inflammation in aged APP(SW) transgenic mice. Neurobiol Aging.

[29] Paradis E, Douillard H, Koutroumanis M, Goodyer C, LeBlanc A (1996). Amyloid beta peptide of Alzheimer’s disease downregulates Bcl-2 and upregulates bax expression in human neurons. J Neurosci.

[30] Smits HA, Rijsmus A, van Loon JH, Wat JW, Verhoef J, Boven LA, Nottet HS (2002). Amyloid-beta-induced chemokine production in primary human macrophages and astrocytes. J Neuroimmunol.

[31] Itzhaki RF, Wozniak MA, Appelt DM, Balin BJ (2004). Infiltration of the brain by pathogens causes Alzheimer’s disease. Neurobiol Aging.

[32] Miklossy J, Kis A, Radenovic A, Miller L, Forro L, Martins R, Reiss K, Darbinian N, Darekar P, Mihaly L, Khalili K (2006). Beta-amyloid deposition and Alzheimer’s type changes induced by *Borrelia* spirochetes. Neurobiol Aging.

[33] **SABiosciences, a QIAGEN company** [http://pcrdataanalysis.sabiosciences.com/pcr/arrayanalysis.php]

[34] Balin BJ, Little CS, Hammond CJ, Appelt DM, Whittum-Hudson JA, Gerard HC, Hudson AP (2008). *Chlamydophila pneumoniae* and the etiology of late-onset Alzheimer’s disease. J Alzheimers Dis.

[35] Rasmussen SJ, Eckmann L, Quayle AJ, Shen L, Zhang YX, Anderson DJ, Fierer J, Stephens RS, Kagnoff MF (1997). Secretion of proinflammatory cytokines by epithelial cells in response to chlamydia infection suggests a central role for epithelial cells in chlamydial pathogenesis. J Clin Invest.

[36] Boelen E, Steinbusch HW, van der Ven AJ, Grauls G, Bruggeman CA, Stassen FR (2007). *Chlamydia pneumoniae* infection of brain cells: an *in vitro* study. Neurobiol Aging.

[37] Carratelli CR, Paolillo R, Rizzo A (2007). *Chlamydia pneumoniae* stimulates the proliferation of HUVEC through the induction of VEGF by THP-1. Int Immunopharmacol.

[38] Gaydos CA, Summersgill JT, Sahney NN, Ramirez JA, Quinn TC (1996). Replication of *Chlamydia pneumoniae in vitro* in human macrophages, endothelial cells, and aortic artery smooth muscle cells. Infect Immun.

[39] Haranaga S, Yamaguchi H, Friedman H, Izumi S, Yamamoto Y (2001). *Chlamydia pneumonia*e infects and multiplies in lymphocytes *in vitro*. Infect Immun.

[40] Gieffers J, van Zandbergen G, Rupp J, Sayk F, Kruger S, Ehlers S, Solbach W, Maass M (2004). Phagocytes transmit *Chlamydia pneumoniae* from the lungs to the vasculature. Eur Respir J.

[41] Koskiniemi M, Gencay M, Salonen O, Puolakkainen M, Farkkila M, Saikku P, Vaheri A (1996). *Chlamydia pneumoniae* associated with central nervous system infections. Eur Neurol.

[42] Little CS, Hammond CJ, MacIntyre A, Balin BJ, Appelt DM (2004). *Chlamydia pneumoniae* induces Alzheimer-like amyloid plaques in brains of BALB/c mice. Neurobiol Aging.

[43] Boman J, Soderberg S, Forsberg J, Birgander LS, Allard A, Persson K, Jidell E, Kumlin U, Juto P, Waldenstrom A, Wadell G (1998). High prevalence of *Chlamydia pneumoniae* DNA in peripheral blood mononuclear cells in patients with cardiovascular disease and in middle-aged blood donors. J Infect Dis.

[44] Griffin WS, Stanley LC, Ling C, White L, MacLeod V, Perrot LJ, White CL, Araoz C (1989). Brain interleukin 1 and S-100 immunoreactivity are elevated in Down syndrome and Alzheimer disease. Proc Natl Acad Sci U S A.

[45] Sheng JG, Ito K, Skinner RD, Mrak RE, Rovnaghi CR, Van Eldik LJ, Griffin WS (1996). In *vivo* and *in vitro* evidence supporting a role for the inflammatory cytokine interleukin-1 as a driving force in Alzheimer pathogenesis. Neurobiol Aging.

[46] Mrak RE, Griffin WS (2001). Interleukin-1, neuroinflammation, and Alzheimer’s disease. Neurobiol Aging.

[47] Serou MJ, DeCoster MA, Bazan NG (1999). Interleukin-1 beta activates expression of cyclooxygenase-2 and inducible nitric oxide synthase in primary hippocampal neuronal culture: platelet-activating factor as a preferential mediator of cyclooxygenase-2 expression. J Neurosci Res.

[48] Griffin WS, Sheng JG, Gentleman SM, Graham DI, Mrak RE, Roberts GW (1994). Microglial interleukin-1 alpha expression in human head injury: correlations with neuronal and neuritic beta-amyloid precursor protein expression. Neurosci Lett.

[49] Blum-Degen D, Muller T, Kuhn W, Gerlach M, Przuntek H, Riederer P (1995). Interleukin-1 beta and interleukin-6 are elevated in the cerebrospinal fluid of Alzheimer’s and de novo Parkinson’s disease patients. Neurosci Lett.

[50] Cacabelos R, Barquero M, Garcia P, Alvarez XA, de Varela SE (1991). Cerebrospinal fluid interleukin-1 beta (IL-1 beta) in Alzheimer’s disease and neurological disorders. Methods Find Exp Clin Pharmacol.

[51] Quintanilla RA, Orellana DI, Gonzalez-Billault C, Maccioni RB (2004). Interleukin-6 induces Alzheimer-type phosphorylation of tau protein by deregulating the cdk5/p35 pathway. Exp Cell Res.

[52] Gadient RA, Otten UH (1997). Interleukin-6 (IL-6) - a molecule with both beneficial and destructive potentials. Prog Neurobiol.

[53] Meylan E, Tschopp J, Karin M (2006). Intracellular pattern recognition receptors in the host response. Nature.

[54] Hollox EJ, Armour JA, Barber JC (2003). Extensive normal copy number variation of a beta-defensin antimicrobial-gene cluster. Am J Hum Genet.

[55] Soscia SJ, Kirby JE, Washicosky KJ, Tucker SM, Ingelsson M, Hyman B, Burton MA, Goldstein LE, Duong S, Tanzi RE, Moir RD (2010). The Alzheimer’s disease-associated amyloid beta-protein is an antimicrobial peptide. PLoS One.

[56] Rosenstiel P, Sina C, End C, Renner M, Lyer S, Till A, Hellmig S, Nikolaus S, Folsch UR, Helmke B, Autschbach F, Schirmacher P, Kioschis P, Hafner M, Poustka A, Mollenhauer J, Schreiber S (2007). Regulation of DMBT1 via NOD2 and TLR4 in intestinal epithelial cells modulates bacterial recognition and invasion. J Immunol.

[57] Shukla SD, Paul A, Klachko DM (1992). Hypersensitivity of diabetic human platelets to platelet activating factor. Thromb Res.

[58] Schroder K, Tschopp J (2010). The inflammasomes. Cell.

[59] Pereira MS, Morgantetti GF, Massis LM, Horta CV, Hori JI, Zamboni DS (2011). Activation of NLRC4 by flagellated bacteria triggers caspase-1-dependent and -independent responses to restrict *Legionella pneumophila* replication in macrophages and *in vivo*. J Immunol.

[60] Masters SL, O’Neill LA (2011). Disease-associated amyloid and misfolded protein aggregates activate the inflammasome. Trends Mol Med.

[61] Chakraborty S, Kaushik DK, Gupta M, Basu A (2010). Inflammasome signaling at the heart of central nervous system pathology. J Neurosci Res.

[62] Franchi L, Eigenbrod T, Munoz-Planillo R, Nunez G (2009). The inflammasome: a caspase-1-activation platform that regulates immune responses and disease pathogenesis. Nat Immunol.

[63] Silverman GA, Bird PI, Carrell RW, Church FC, Coughlin PB, Gettins PG, Irving JA, Lomas DA, Luke CJ, Moyer RW, Pemberton PA, Remold-O’Donnell E, Salvesen GS, Travis J, Whisstock JC (2001). The serpins are an expanding superfamily of structurally similar but functionally diverse proteins. Evolution, mechanism of inhibition, novel functions, and a revised nomenclature. J Biol Chem.

[64] Law RH, Zhang Q, McGowan S, Buckle AM, Silverman GA, Wong W, Rosado CJ, Langendorf CG, Pike RN, Bird PI, Whisstock JC (2006). An overview of the serpin superfamily. Genome Biol.

[65] Conductier G, Blondeau N, Guyon A, Nahon JL, Rovere C (2010). The role of monocyte chemoattractant protein MCP1/CCL2 in neuroinflammatory diseases. J Neuroimmunol.

[66] Galimberti D, Schoonenboom N, Scheltens P, Fenoglio C, Bouwman F, Venturelli E, Guidi I, Blankenstein MA, Bresolin N, Scarpini E (2006). Intrathecal chemokine synthesis in mild cognitive impairment and Alzheimer disease. Arch Neurol.

[67] Fiala M, Zhang L, Gan X, Sherry B, Taub D, Graves MC, Hama S, Way D, Weinand M, Witte M, Lorton D, Kuo YM, Roher AE (1998). Amyloid-beta induces chemokine secretion and monocyte migration across a human blood-brain barrier model. Mol Med.

[68] Yamamoto M, Horiba M, Buescher JL, Huang D, Gendelman HE, Ransohoff RM, Ikezu T (2005). Overexpression of monocyte chemotactic protein-1/CCL2 in beta-amyloid precursor protein transgenic mice show accelerated diffuse beta-amyloid deposition. Am J Pathol.

